# Surveillance of tobacco retail density in Beirut, Lebanon using electronic tablet technology

**DOI:** 10.1186/1617-9625-12-3

**Published:** 2014-02-17

**Authors:** Ramzi G Salloum, Rima T Nakkash, Allison E Myers, Jan M Eberth, Kathryn A Wood

**Affiliations:** 1 Department of Health Services Policy & Management, Arnold School of Public Health, University of South Carolina, 915 Greene Street, Suite 351, Columbia, SC 29208, USA; 2 Department of Health Promotion and Community Health, Faculty of Health Sciences, American University of Beirut, Beirut, Lebanon; 3 Department of Health Behavior, Gillings School of Global Public Health, University of North Carolina at Chapel Hill, Chapel Hill, USA; 4 Department of Epidemiology and Biostatistics, Arnold School of Public Health, University of South Carolina, Columbia, USA; 5 School of Nursing, Duke University, Durham, USA

## Abstract

**Background:**

Lebanon has alarmingly high rates of tobacco use. The objective of this study is to examine the density of tobacco retail outlets and distance to schools as well as to survey retail pricing in a large district of Beirut, Lebanon.

**Findings:**

We observed 100 tobacco retail outlets and collected data using iPad® technology. Store locations were recorded with Global Positioning System coordinates. The distances between all pairs of tobacco retail outlets and all schools were calculated. For 52% of tobacco outlets, the nearest distance to other tobacco retail outlets was < 50 meters and 17% were within a 100-meter radius of a school. We found a high proportion of tobacco retailers with close proximity to schools. The overall retailer density was 1.25 stores per 1,000 people.

**Conclusions:**

These findings call for additional regulation including the establishment of strict density standards.

## Introduction

The tobacco epidemic is a growing phenomenon in Lebanon and the greater Arab region in part due to a weak public health regulatory environment [1]. The failure of public policy in responding to this epidemic has allowed the multinational tobacco industry nearly free rein for product marketing and promotion [2-4]. Despite a bleak outlook, glimmers of hope appeared over the past few years as Lebanon ratified the Framework Convention on Tobacco Control (FCTC) in 2005 and adopted its first comprehensive tobacco control policy measures in 2012 [5]. These measures included a ban on all forms of advertising and sponsorship, effective March 2012, and a ban on indoor smoking in public places, effective September 2012.

Given the lack of strong surveillance systems, few studies have examined the tobacco regulatory environment in Lebanon or the Arab region [1]. To our knowledge, only one previous study has reported on tobacco point-of-sale (POS) in the region, describing POS tobacco advertising and marketing, and POS industry activity [5]. The aim of this study was to document tobacco retail outlet density, measured as the number of retailers per 1000 population, proximity of tobacco retailers to schools, and product pricing with the use of a novel electronic tablet tool.

## Methods

### Sample

We conducted observational store audits in *Ras Beirut*, a diverse mixed-use district of the capital city that is adjacent to the American University of Beirut. This high-income neighborhood (approximately 5 km^2^ in area and population of 80,000 [6]) is considered a main cultural and intellectual center within the city. The research area comprises 10 city sectors; printed maps of each sector were generated using Google Maps. After receiving a half-day training session, five university students canvassed the entire district with predetermined routes, marking the location of each store and assigning it a unique identification number, thus creating a census of all tobacco retail outlets (n = 100) in the *Ras Beirut* district. Next, the students conducted the interior and exterior audits and electronically recorded their observations. After excluding 3 stores that did not sell tobacco, 100 store audits were completed in June 2012. Tobacco retail outlets observed were all stores that sold cigarettes including small, convenience stores or mini markets; tobacco or liquor stores; bakeries; gas stations; and large or super markets. Additional details of our sampling strategy are reported elsewhere [5].

### Measures

Store audit items were designed to solicit information on POS advertising, placement of tobacco products within each store, and advertised price per pack of *Marlboro* (top-selling premium brand) and *Cedars* (local/value brand). Our findings on product advertisement and placement are reported elsewhere [5]. The store audit data and Global Positioning System (GPS) coordinates of the stores were recorded into a web form using internet-enabled tablet technology (iPad®). We used the ‘*Where Am I At?*’ © application (app) for iPhone®/iPad® to capture GPS coordinates.

### Data analysis

Tobacco retailer density was calculated by dividing the number of stores by the area population and converted to a measure of the number of retailers per 1000 population. The point distance tool in ArcGIS (version 10, ESRI, Redland, CA, USA) was used to calculate the (1a) straight-line distance between each tobacco retail outlet and the next nearest tobacco retail outlet; (1b) the numbers of other tobacco retail outlets within a 50, 100, 200 and 400 meter radius of each outlet; and (2) straight line distance from each school to the nearest tobacco retail outlet as well as the numbers of tobacco retail outlets within a 100- and 200-meter radius of each school. We used GPS coordinates to generate a map of all retail outlets in Google Maps ©. Descriptive statistics were calculated using SAS software (version 9.3, SAS Institute Inc., Cary, NC, USA).

## Results

**Figure 1:**
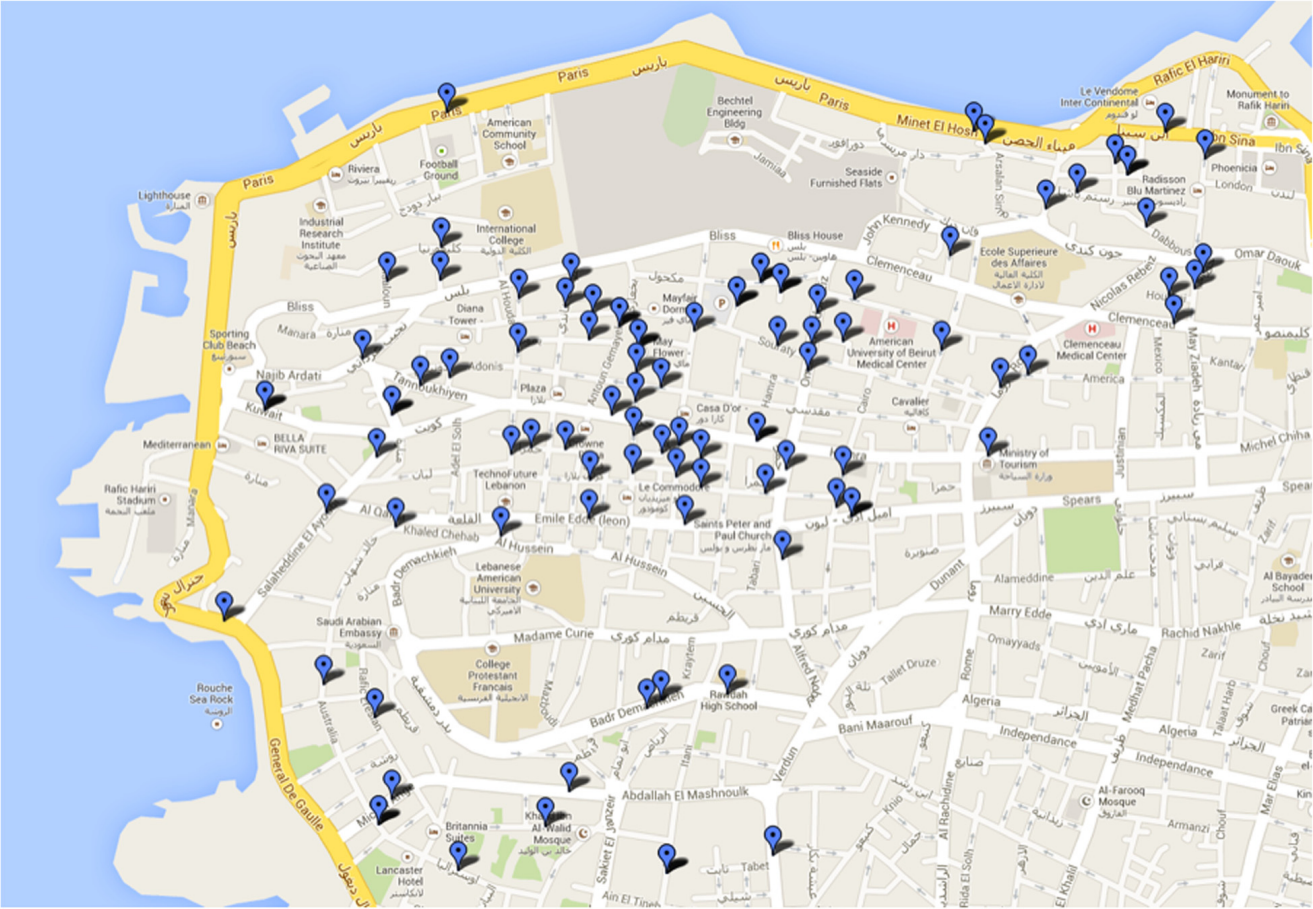
Map of tobacco retailers in Ras Beirut.

See Figure 1 for the distribution of retail outlets across the area. Of the retail outlets surveyed in *Ras Beirut*, 90% were small convenience stores or mini markets. Our audit results (Table 1) confirmed price fixing in the Lebanese market for cigarettes: the price per pack of *Marlboro* was the same at all audited stores, 2500 Lebanese Pounds (approximately $1.67), and the price per pack for the local *Cedars* brand was 750 Lebanese Pounds (approximately $0.50). The nearest tobacco retail outlet was within a 50-meter radius for 52% of stores and within a 100-meter radius for 83% of stores. We found that 17% of stores were within 100 meters of a school and 38% within a 200-meter radius. This corresponds to approximately 1.25 locations per 1,000 people (1.60 locations per 1,000 people aged 15+, and 5.68 locations per 1,000 children <15).

**Table 1:** Price per pack and tobacco retail density

	**Store audits (n = 100)**
Store type (%)	
Large store or supermarket	6
Small grocery or convenience store, mini market	90
Other	4
Price per pack in Lebanese Pounds (and USD equivalent)	
Marlboro	2500 ($1.67)
Cedars	750 ($0.50)
Retailer density	
Stores per 1,000 (age 15+)	1.60
Stores per 1,000 (age <15)	5.68
Stores per 1,000 (all ages)	1.25
Clustering of tobacco retail outlets (%)	
≥ 1 other store(s) within 50 m radius	52
≥ 1 other store(s) within 100 m radius	83
≥ 1 other store(s) within 200 m radius	96
≥ 1 other store(s) within 400 m radius	100
Proximity to schools (%)	
Stores within 100 m radius of school	17
Stores within 200 m radius of school	38

## Discussion

This was the first study in Lebanon and the region to assess several dimensions of POS tobacco sales, including pricing and proximity to schools. Further, our team employed an innovative approach for data collection. This is one of only a handful of POS audits to rely on electronic mobile input devices [7]. In addition to a lack of compliance with the recent national advertising ban on tobacco products [5], our study found significant clustering of retailers, high retailer density (1.25/1000 population), and a high proportion of tobacco retailers located within close proximity (<200 m) to schools. Our findings of high tobacco retailer density were similar to results from audits conducted in the United States, Canada, China, India, and Guatemala [8-13]. The retail environment remains the least regulated channel of tobacco sales marketing worldwide and little is known on the potential impact of such regulation [14]. In a region with a growing tobacco epidemic and a lack of regular surveillance channels, more research is needed on the tobacco retail environment. Our study was the first in Lebanon and the region to measure tobacco retailer density and proximity of tobacco retail outlets to schools. While an advertising ban has been implemented in Lebanon, the regulations did not establish density standards for tobacco retailers. These findings call for additional regulation to limit the locations, number, type, and density of tobacco retailers.

## Competing interests

Ms. Myers has participated in the development of a mobile store audit data collection system and tobacco retailer mapping system that will generate royalties when licensed. Ms. Myers is the Deputy Director of Counter Tools, a nonprofit organization with a mission to disseminate store audit and mapping tools for tobacco control.

## Authors’ contributions

The study was jointly designed by RS, RN, AM, and KW. AM provided the data collection software/tool. RS, KW, and RN led the data collection. RS and AM were responsible for data analysis. Findings were jointly interpreted by all authors. All authors contributed to successive drafts. The final manuscript was approved by all authors.
